# Supplementary material for: Structural and functional analyses of SARS-CoV-2 Nsp3 and its specific interactions with the 5’ UTR of the viral genome
Source: Microbiol Spectr. 2025 Jul 7;13(8):e02871-24. doi: 10.1128/spectrum.02871-24 (PMC12323374; doi:10.1128/spectrum.02871-24)
Supplement: Supplemental figures and tables — Fig. S1 to S6 and Table S1. [file spectrum.02871-24-s0001.pdf]

Supplemental Data Figure 1

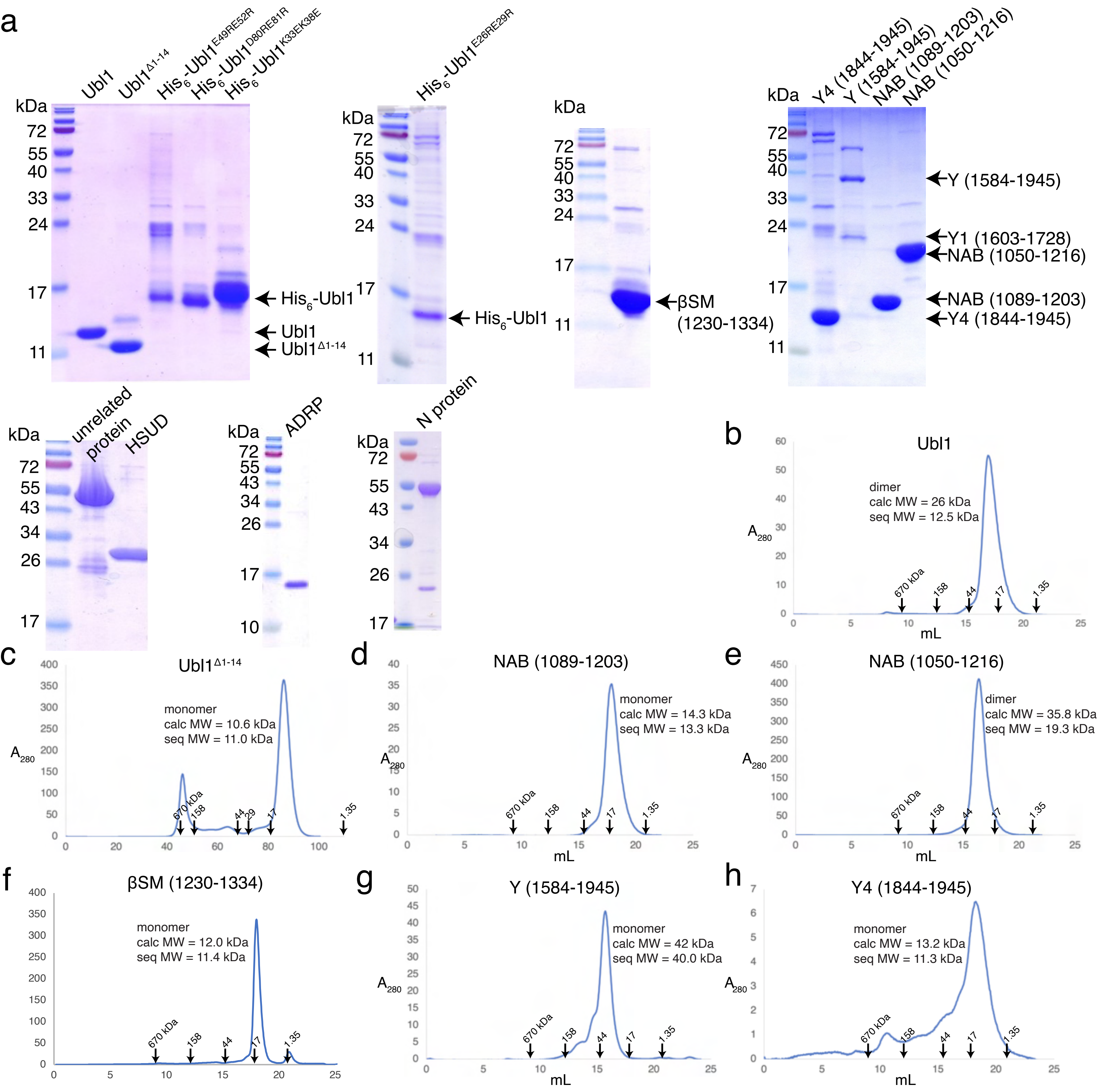

Supplemental Data Figure 2

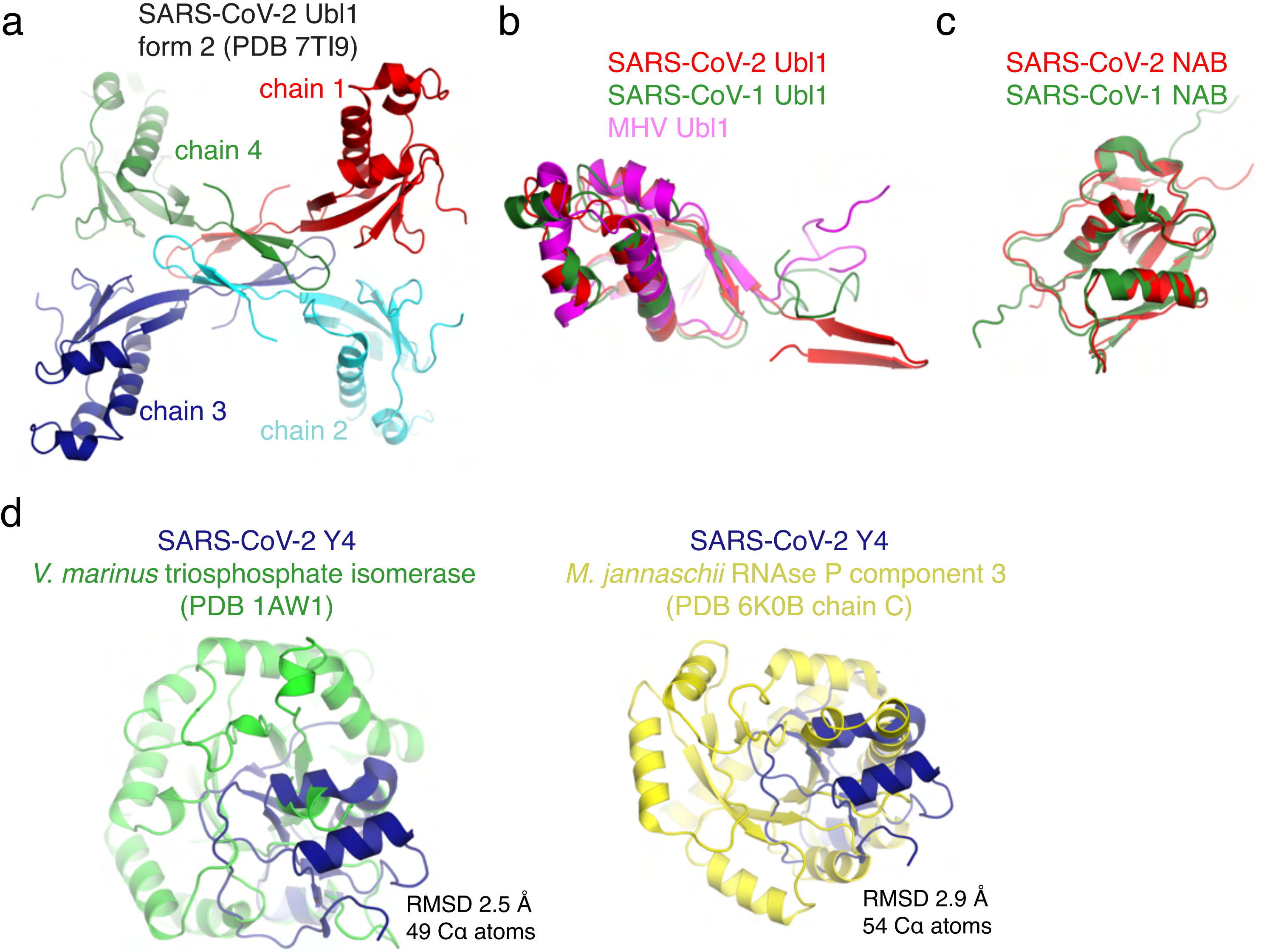

Supplemental Data Figure 3

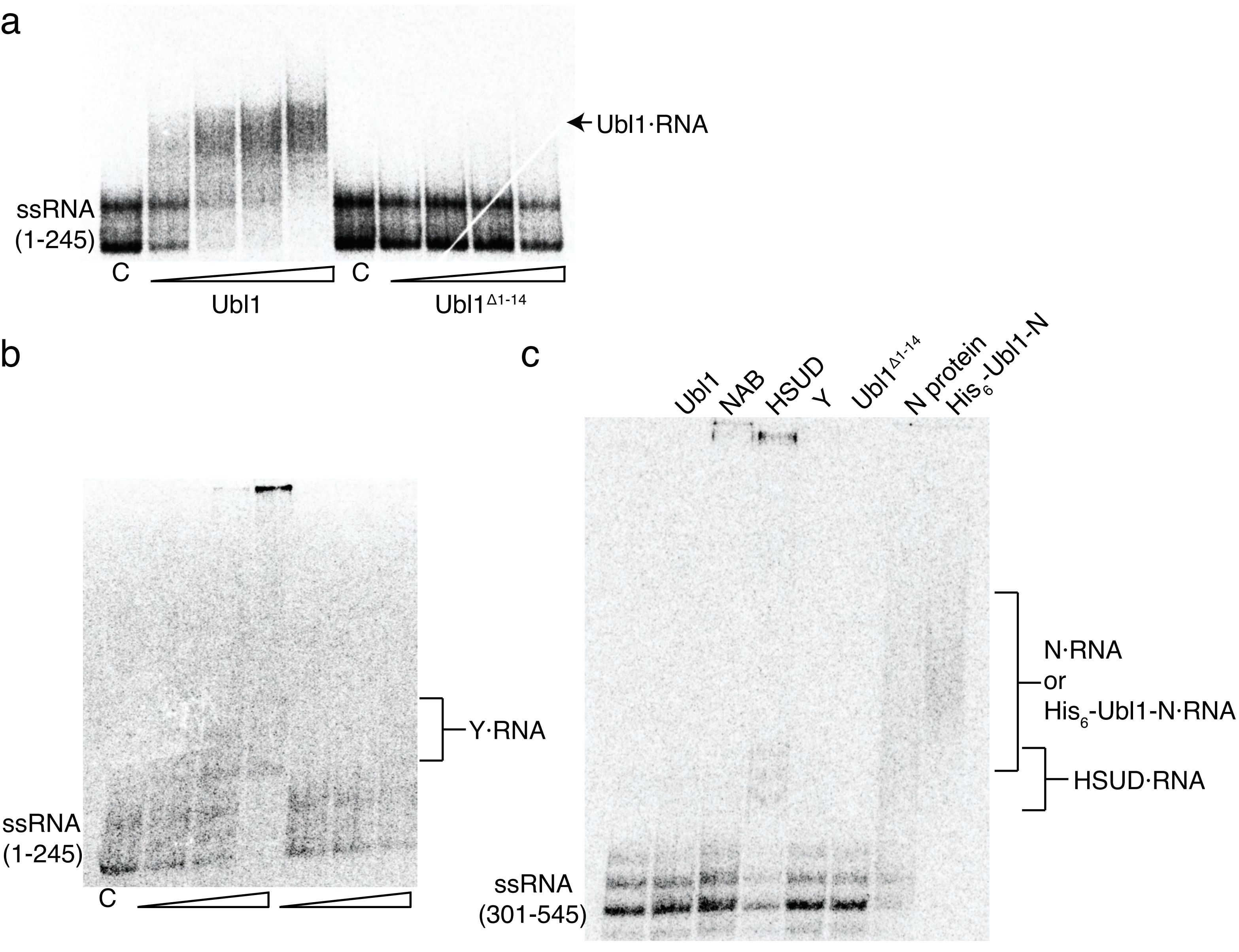

Supplemental Data Figure 4

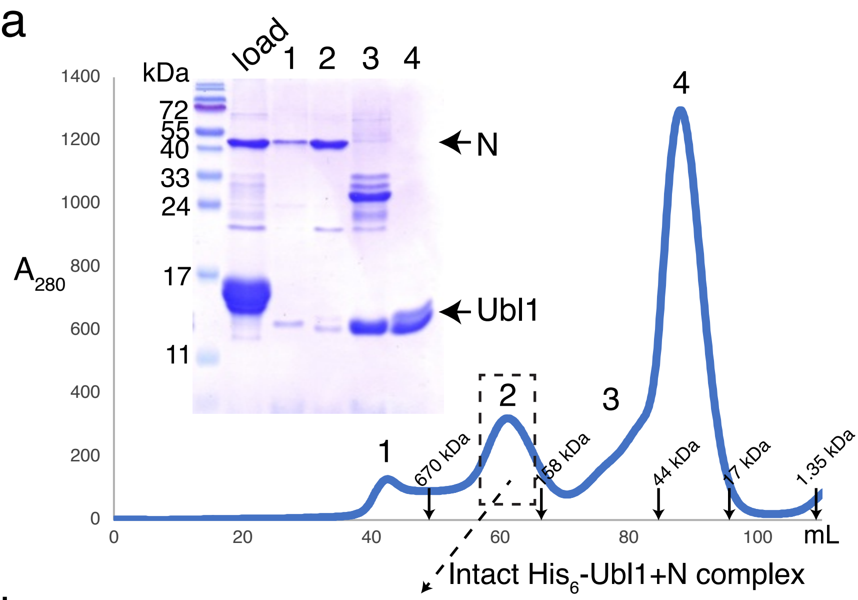

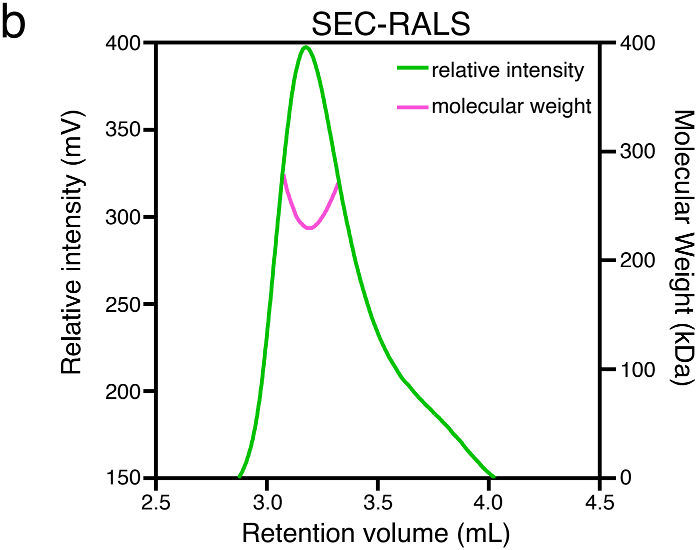

**Molecular weight (kDa)**	**Radius of hydration R_h (nm)**	**Radius of gyration R_g (nm)**	**Recovery of Sample (%)**
248,369	7.015	39.71	96.40

Supplemental Data Figure 5

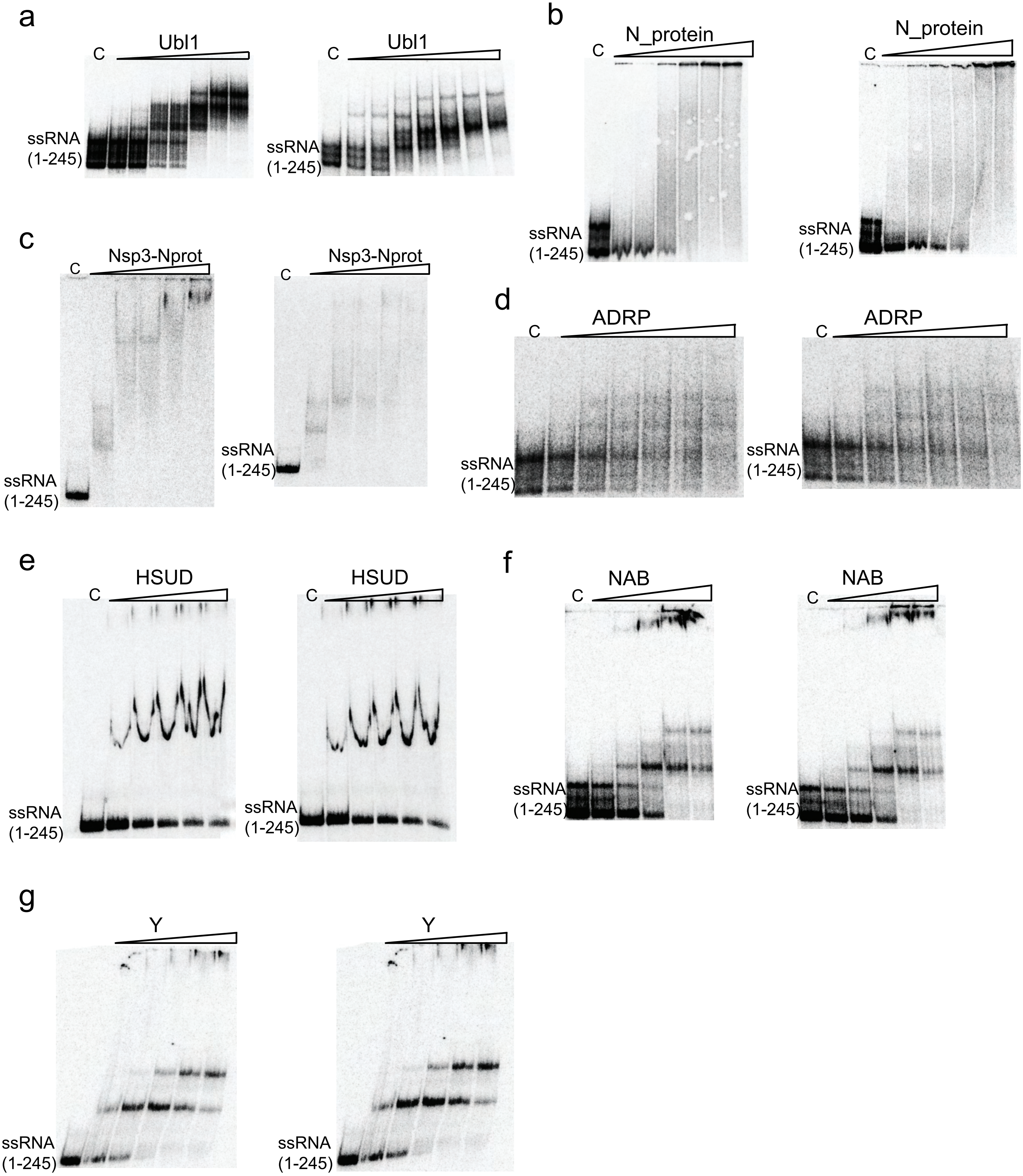

Supplemental Data Figure 6

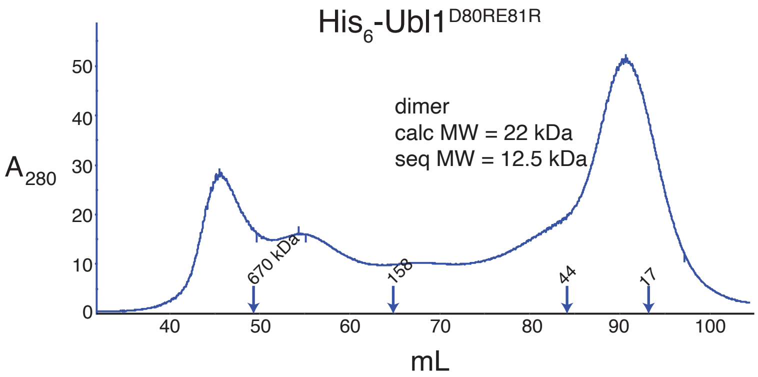

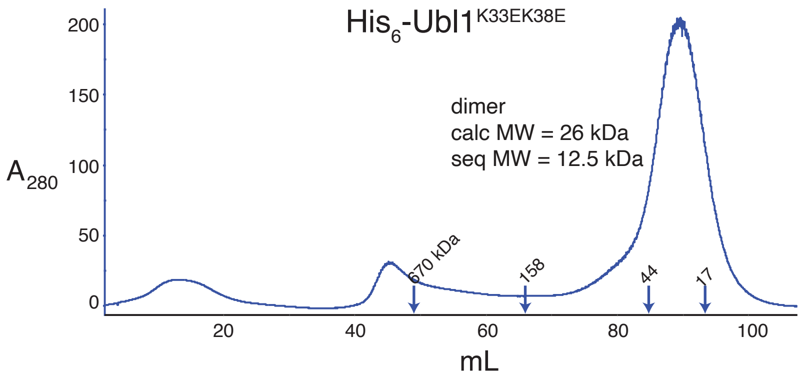

Supplemental Data Figure Legends

1. a) SDS-PAGE gels showing purified Nsp3 domains and N protein used for crystallization or RNA binding assays throughout the study. b through h) Size exclusion chromatography profiles of purified Nsp3 domains used in the study.

2. Higher-order oligomerization observed through crystal symmetry in the Nsp3 Ubl1 form 2 crystal structure (PDB 7TI9) determined in this study. b) Comparison of the structures of SARS-CoV-2 Nsp3 Ubl1, SARS-CoV Nsp3 Ubl1 and MHV Ubl1. c) Comparison of the structures of SARS-CoV-2 Nsp3 NAB and SARS-CoV Nsp3 NAB domains. d) Overlay of the SARS-CoV-2 Nsp3 Y4 region structure with that of a triosphosphate isomerase (PDB 1AW1). e) Overlay of the SARS-CoV-2 Y4 region structure with that of a RNAse P component 3 (PDB 6K0B).

3. a) EMSA showing binding activity of 32 – 80 μM Nsp3 Ubl1 (left side) and 64 – 160 μM Ubl1$^{\Delta 1\text{-}14}$ (right side) to the 5' UTR (1-245) ssRNA. b) EMSA showing binding activity of 1.8 – 7 μM of either the full Nsp3 Y domain (left side) or Y3 region (right side) to the 5' UTR (1-245) c) EMSA evaluating binding to the 5' UTR (301-545) region, by 80 μM Ubl1, 400 μM NAB, 100 μM HSUD, 5 μM Y, 80 μM Ubl1$^{\Delta 1\text{-}14}$, 38 μM N protein, and 80 nM His_6-Ubl1-N protein complex. Only HSUD and N protein interacted with this ssRNA.

4. Preparation of His_6-Ubl1-N protein complex. a) SDS-PAGE showing load = mixture of His_6-Ubl1 and N proteins, and 4 fractions from size exclusion chromatography profile shown below. Fraction 2 corresponded to His_6-Ubl1-N protein complex used for RNA binding studies. b) SEC-RALS results for His_6-Ubl1-N protein complex, showing a complex with molecular weight 248 kDa, which we interpreted as a 4:4 ratio of His_6-Ubl1 to N protein.

5. ssRNA binding by Nsp3: replicate EMSA gels (for results in Figure 2 (a-c) and Figure 3 (d-g). Binding of 7 – 287 μM Ubl1 (a), 313 nM - 3.1 μM N protein (b), 69 - 230 nM Ubl1-N complex (c), 80-550 μM ADRP (d), 20 – 197 μM HSUD (e), 51 μM - 1 mM NAB (f), and 1.1 – 7.1 μM Y domain (g) to 5'-UTR (1-245) RNA . The results are also shown as a mean ± standard deviation of these EMSA gels in Fig. 2 and Fig3. 'C' indicates no enzyme control (in all gels).

6. Size exclusion chromatography profiles of two selected Ubl1 variants used in the study.

Supplemental Data Table 1. X-ray crystallographic statistics.

Structure	Nsp3 Ubl1 form 1	Nsp3 Ubl1 form 2	Nsp3 NAB
PDB code	7KAG	7TI9	7LGO
Data collection			
Space group	C2	F222	$P6_522$
Cell dimensions			
a, *b*, *c* (Å)	81.83, 147.57, 70.14	84.27, 111.76, 145.99	76.67, 76.67, 204.15
α, β, γ, (°)	90, 125.9, 90	90, 90, 90	90, 90, 120
Resolution, Å	50.00 – 3.13	30.00 – 2.73	25.0 – 2.44
R_{merge}[a]	0.053 (0.933)[*]	0.063 (0.576)	0.014 (1.35)
R_{pim}[b]	0.035 (0.608)	0.024 (0.270)	0.04 (0.386)
$CC_{1/2}$[*]	0.612	0.818	0.633
I / σ (I)	24.5 (1.04)	29.2 (1.36)	17.7 (1.36)
Completeness, %	96.0 (83.5)	98 (80.9)	99.9 (99.1)
Redundancy	3.3 (2.8)	7.7 (4.7)	11.4 (10)
Refinement			
Resolution, Å	35.78 – 3.21	27.91 – 2.73	24.91 – 2.45
No. unique reflections: working, test	8008, 793	9242, 463	12684, 1022
R_{work}/R_{free}[c]	21.4/24.8 (32.6/42.5)	22.2/24.9 (32.1/39.7)	26.1/31.8 (35.9/41.8)
No. atoms			
Protein	1750	879	1812
Solvent	57	15	N/A
Water	22	36	142
B-factors			
Protein	54.2	84.0	67.2
Solvent	56.2	94.4	N/A
Water	19.2	72.5	44.8
R.m.s. deviations			
Bond lengths, Å	0.002	0.002	0.004
Bond angles, °	0.512	0.502	0.838
Ramachandran plot			
Favored, %	93.5	94.5	92.2
Allowed, %	6.5	5.5	7.8
Outliers, %	0	0	0

**Structure**	**Nsp3 βSM**	**Nsp3 Y4**
PDB code	7T9W	7RQG
Data collection		
Space group	$P4_3$	P1
Cell dimensions		
a, *b*, *c* (Å)	157.46, 157.46, 79.62	41.59, 49.14, 54.63
α, β, γ, (°)	90, 90, 90	80.22, 89.65, 77.94
Resolution, Å	30.00 – 2.20	30.00 – 2.17
R_{merge}[a]	0.104 (0.893)	0.104 (0.661)
R_{pim}[b]	0.04 (0.389)	0.073 (0.487)
$CC_{1/2}$[*]	0.658	0.546
$I / \sigma (I)$	17.4 (1.23)	15.26 (1.64)
Completeness, %	100 (99.9)	90.5 (89.2)
Redundancy	7.5 (6.1)	2.9 (2.7)
Refinement		
Resolution, Å	29.42 – 2.20	28.05 – 2.17
No. unique reflections: working, test	94456, 1875	19491, 975
R_{work}/R_{free}[c]	26.2/31.6 (37.6/40.2)	19.7/24.2 (25.5/30.6)
No. atoms		
Protein	10879	2995
Solvent	13	N/A
Water	991	257
B-factors		
Protein	56.8	50.9
Solvent	62.5	N/A
Water	47.8	56.7
R.m.s. deviations		
Bond lengths, Å	0.003	0.002
Bond angles, °	0.535	0.453
Ramachandran plot		
Favored, %	96.4	99.7
Allowed, %	3.6	0.3
Outliers, %	0	0

[*]All values in brackets and $CC_{1/2}$ values refer to highest resolution shells.

[a]$R_{merge} = \Sigma_{hkl}\Sigma_j|I_{hkl.j}\text{-}I_{hkl}|/\Sigma_{hkl}\Sigma_j I_{hk,j}$, where $I_{hkl,j}$ and $\langle I_{hk,j} \rangle$ are the *j*th and mean measurement of the intensity of reflection *j*.
[b]$R_{pim} = \Sigma_{hkl}\sqrt{(n/n\text{-}1)}\ \Sigma^{n}_{j=1}|I_{hkl.j}\text{-}I_{hkl}|/\Sigma_{hkl}\Sigma_j I_{hk,j}$
[c]$R = \Sigma|F_p^{obs} - F_p^{calc}|/\Sigma F_p^{obs}$, where F_p^{obs} and F_p^{calc} are the observed and calculated structure factor amplitudes, respectively.
N/A = not applicable.
